# Nursing home admission after myocardial infarction in the elderly: A nationwide cohort study

**DOI:** 10.1371/journal.pone.0202177

**Published:** 2018-08-15

**Authors:** Laerke Smedegaard, Kristian Kragholm, Anna-Karin Numé, Mette Gitz Charlot, Gunnar Hilmar Gislason, Peter Riis Hansen

**Affiliations:** 1 Herlev & Gentofte University Hospital, Department of Cardiology, Hellerup, Denmark; 2 Aalborg University Hospital, Department of Clinical Epidemiology and Biostatistics, Aalborg, Denmark; 3 The Institute of Public Health, University of Southern Denmark, Copenhagen, Denmark; 4 Faculty of Health & Medical Sciences, University of Copenhagen, Copenhagen, Denmark; 5 The Danish Heart Foundation, Copenhagen, Denmark; University of Antwerp, BELGIUM

## Abstract

**Background:**

Data on nursing home admission after myocardial infarction (MI) in the elderly are scarce. We investigated nursing home admission within 6 months and 2 years after MI including predictors for nursing home admission in a nationwide cohort of elderly patients.

**Methods:**

Using Danish nationwide registries, we identified all subjects 65 years or older residing at home who were discharged following first-time MI in the period 2008–2015. We determined sex- and age-stratified incidence rates per 1000 person years (IRs) and incidence rate ratios (IRRs) of nursing home admissions using Poisson regression models compared to the Danish population 65 years or older with no prior MI. Poisson regression models were also applied to identify predictors of nursing home admission.

**Results:**

The 26,539 patients who were discharged after MI had a median age of 76 (quartile 1-quartile 3: 70–83) years. The IRs of nursing home admission after MI increased with increasing age and for 80-84-year-old women IRs after 6 months and 2 years were 113.9 and 62.9, respectively, compared to 29.4 for women of the same age with no prior MI. The IRs for 80-84-year-old men after MI were 56.0 and 36.2, respectively, compared to 24.3 for men of the same age with no prior MI. In adjusted analyses the 6 months and 2 years IRRs for 80-84-year-old subjects were 2.56 (95% CI 2.11–3.10) and 1.41 (95% CI 1.22–1.65) for women and 1.74 (95% CI 1.34–2.25) and 1.05 (95% CI 0.88–1.26) for men, respectively. Predictors were advanced age, dementia, home care, Parkinson’s disease, cerebrovascular disease, living alone, depression, and arrhythmia.

**Conclusion:**

In elderly patients discharged following first-time MI, the risk of subsequent nursing home admission within 6 months was 2-fold higher compared to an age-stratified population with no prior MI. After 2 years this risk remained higher in women.

## Introduction

Myocardial infarction (MI) is a leading cause of death and disability worldwide and the risk remains highest among the elderly [1–3]. However, life expectancy is increasing and patients admitted with MI are getting older and have more comorbidities [4]. Also, awareness of guideline-recommended treatment of MI in elderly and subjects with frailty [5,6], including considerations regarding the use of invasive procedures, is increasing and in-hospital mortality of elderly MI patients has declined [6]. Nonetheless, survival with highly impaired daily functioning is, of course, not an optimal outcome.

A substantial part of survivors of MI experience a significant decline in physical function or loss of independence by one year after MI [7,8] and among the greatest fears in the elderly are the loss of independence and nursing home admission [9]. Therefore, knowledge about the care dependency, quality of life and physical function after MI is of importance for continued development of strategies to counteract negative consequences of MI on these variables.

Notably, three quarters of patients admitted to nursing homes have been hospitalised 6 months prior to admission [10]. In the general population predictors of nursing home admission are well described and include advanced age, cognitive dysfunction, poor social support, physical disability, and depression [11–16]. One study reported that MI was one of many predictors of nursing home admission in a cohort of elderly without dementia [15]. In addition, another study has shown that patients with MI residing in nursing homes were less likely to receive reperfusion therapy compared to matched community dwelling patients [17], but nursing home admission after MI has not been investigated in detail.

Admission to a nursing home represents a considerable change in a person’s life and is associated with great psychological concerns [9]. Furthermore it is associated with societal costs, especially with an increasing proportion of elderly and elderly with disabilities and comorbidities [18]. For decades patients with MI have experienced increasing likelihood of survival [19], but subsequent morbidity and frailty have not necessarily accompanied this favourable development. In fact, ischemic heart disease is the leading contributor to the burden of disease in people aged 60 years or older [20]. Identifying MI patients at high risk of admission to nursing homes may be the first step for designing targeted and personalised rehabilitation programs to keep patients able and fit to live in own homes after post-MI rehabilitation. Therefore we conducted a nationwide registry-based study that aimed to investigate nursing home admissions 6 months and 2 years after MI and additionally we aimed to identify predictors of nursing home admission.

## Methods

### Setting

The study was conducted in Denmark where all 5.6 million citizens are covered by publicly financed national health insurance, which ensures equal access to healthcare services free of personal charge. The Danish administrative registries hold a wide range of health related variables and cross-linkage of information is possible due to a unique and permanent personal identification number [21].

### Study population

This study is a nationwide registry-based cohort study, between January 1, 2008 and December 31, 2015. The study population comprised all Danish residents aged 65 years or older, who were living at home and with no history of MI at time of inclusion. All individuals were included at day of study start or when they turned 65. Of these subjects, we identified a cohort of patients discharged with a first-time primary diagnosis of MI (International Classification of Diseases [ICD] 10^th^ revision code I21) during the study period.

### Data sources

The Danish Civil Registration System holds information on birth, sex and death status and the Danish National Patient Registry contains information on all hospital admissions since 1978 [22,23]. At discharge or after an outpatient visit all contacts are registered by one primary diagnosis, and supplementary secondary diagnoses if appropriate, according to ICD codes [23]. The MI diagnosis has previously been validated in this registry with a sensitivity of 97% [24].

Information on all prescriptions dispensed from pharmacies in Denmark since 1995 is collected in the Danish Register of Medicinal Product Statistics and each drug is classified according to the Anatomic Therapeutical Chemical (ATC) classification. The Danish health care system provides partial reimbursement of drug expenses which ensures a high validity of the registry [25]. Data on living status, personal income, nursing home admission, and home care were provided by Statistics Denmark. Data on living status (living alone or not) and personal income were available on annual basis and we indexed income based on 2015 values [26]. In Denmark, nursing homes have gradually been substituted by nursing apartments and eligibility criteria for these complexes are similar to those of nursing homes; thus we did not distinguish between these facilities and both were captured by the method used in this paper and referred to as ‘nursing homes’ [27–29]. Information on nursing home admissions was collected using a validated method based on addresses and linkage to personal address information from the Danish Civil registry [29]. All citizens are entitled to home care by municipal or private providers when the need is established at a meeting between the citizen and municipal staff and we used the date of these meetings to adjudicate the onset of home care need.

### Main outcome

The main outcome was defined as admission to nursing home. The study population was followed until date of nursing home admission, date of emigration, date of death, or end of study period, i.e., December 31, 2015.

### Covariates

The covariates in the multivariable analyses were selected prior to analysis based on current knowledge and included sex, age, living alone, baseline income, home care, comorbidities (heart failure, arrhythmia, diabetes mellitus, cerebrovascular disease, chronic kidney disease, dementia, Parkinson’s disease, and depression), and calendar year. Comorbidity status at baseline was examined based on discharge and outpatient diagnoses up to five years prior to the date of inclusion. The validity of the diabetes, dementia, and depression diagnoses were enhanced by use of claimed prescriptions for anti-diabetic, anti-dementia, and anti-depressive drugs six month prior to inclusion, respectively. Baseline income was divided into tertiles based on income for individuals aged 65 years or older in the Danish population (first tertile < €29,219; second tertile between € 29,219 and € 44,115; third tertile > € 44,115).

In multivariable analyses within the MI cohort coronary revascularisation (percutaneous coronary intervention [PCI] and coronary artery by-pass grafting [CABG]) from the date of hospitalisation until follow-up were included. To identify hypertension we used treatment with at least two standard antihypertensive agents within a period of 90 days or an ICD code of hypertension [30]. All codes used for definitions of comorbidity and pharmacotherapy are found in S1 Table.

### Statistical analysis

Main outcome measurements were crude age-stratified incidence rates per 1000 person-years (IRs) and adjusted age-stratified incidence rate ratios (IRRs) of nursing home admission after 6 months and 2 years for patients discharged following MI compared to the population 65 years or older with no prior MI. Descriptive characteristics are presented at time of study start for the population with no prior MI and at time of discharge following MI for the MI population, respectively. Time-dependent multivariable Poisson regression models were used to estimate age-stratified IRRs of nursing home admission for MI patients compared to the population with no prior MI. We tested for interactions by inclusion of interaction terms in the model using likelihood ratio tests between sex and age and stratified the model on sex as interactions were present. Furthermore, the models were adjusted for calendar year, living alone, home care, baseline income, along with comorbidity. Using the Lexis diagram principle individuals were allowed to contribute risk-time to both unexposed and exposed groups by splitting the risk-time of each individual into several observations, each combination defined by the time-dependent variables [31]. The time scales included were calendar year (2-year intervals) and age (5-year intervals). Due to splitting of risk-time the risk of nursing home admission in the population with no prior MI remained constant from time of inclusion.

To identify predictors of 6 months nursing home admission in patients with MI, we also applied Poisson regression model. For sex and age estimates we included the before-mentioned variables and added PCI and CABG. In order to evaluate other estimates, age was modelled as a restricted cubic spline.

In a sensitivity analysis we investigated the use of home care after discharge following MI in patients who did not receive home care prior to discharge by use of the Aalen-Johansen estimator accounting for competing risks of death and nursing home admission [32]. Furthermore, admission to nursing home was examined using the same approach. Statistical analyses were performed using SAS version 9.4 (SAS Institute Inc., Gary, NC, USA) and R version 3.2.3 (R Foundation for Statistical Computing, Vienna, Austria).

### Ethics

This study was approved by the Danish Data Protection Agency (local reference No. 2007-58-0015/GEH-2014-014 I-suite 02732). In Denmark, ethical approval is not required for retrospective registry-based studies using de-identified data.

## Results

During the study period we identified 1,369,257 subjects aged 65 years or older. Patients were excluded if they had a history of MI, were living in their own homes at time of inclusion, or if data were missing. The study cohort was comprised of 1,257,851 individuals and among these 26,539 were discharged after first-time MI during follow up. These patients had a median age of 76 (quartile 1-quartile 3: 70–83) years and 15,150 (58.4%) were men. Baseline characteristics are presented in Table 1 for the whole study population and baseline characteristics for the MI population stratified according to sex and age are shown in S2 Table.

**Table 1:** Baseline characteristics of study population.

Variables	Population with no prior MI	MI patients
n	1,231,312	26,539
Age (median [Q1-Q3])	67 (65, 75)	76 (70, 83)
Age group (n [%])		
65–74 years	907,662 (73.7)	11,594 (43.7)
75–84 years	237,895 (19.3)	9907 (37.3)
≥85 years	85,755 (7.0)	5038 (19.0)
Men (%)	549,002 (44.6)	15,510 (58.4)
Living alone (%)	560,092 (45.7)	12,993 (49.0)
Home care (%)	139,175 (11.3)	7425 (28.0)
Income (%)		
First tertile	405,583 (32.9)	13,699 (51.6)
Second tertile	411,147 (33.4)	8138 (30.7)
Third tertile	4145,82 (33.7)	4702 (17.7)
Heart failure (%)	28,263 (2.3)	4675 (17.6)
Arrhythmia (%)	69,025 (5.6)	5052 (19.0)
Hypertension (%)	374,466 (30.4)	14,429 (54.4)
Diabetes (%)	105,141 (8.5)	4574 (17.2)
Chronic kidney disease (%)	12,989 (1.1)	1377 (5.2)
Cerebrovascular disease (%)	62,128 (5.0)	2718 (10.2)
Peripheral artery disease (%)	15,771 (1.3)	1257 (4.7)
COPD (%)	44,588 (3.6)	2913 (11.0)
Dementia (%)	17,265 (1.4)	622 (2.3)
Depression (%)	127,648 (10.4)	3367 (12.7)
Parkinson’s disease (%)	4636 (0.4)	156 (0.6)
Cancer (%)^a^	83,719 (6.8)	2889 (10.9)
Revascularisation during index hospitalisation		
PCI (%)		11,613 (43.8)
CABG (%)		709 (2.7)

MI; Myocardial infarction, Q1-Q3; Quartile 1-quartile 3, COPD; Chronic obstructive lung disease, PCI; Percutaneous coronary intervention, CABG; Coronary artery by-pass grafting.

^a^ Cancer was either active or prior disease; it was diagnosed within 5 years prior to inclusion or MI.

### Risk of nursing home admission

**Fig 1:**
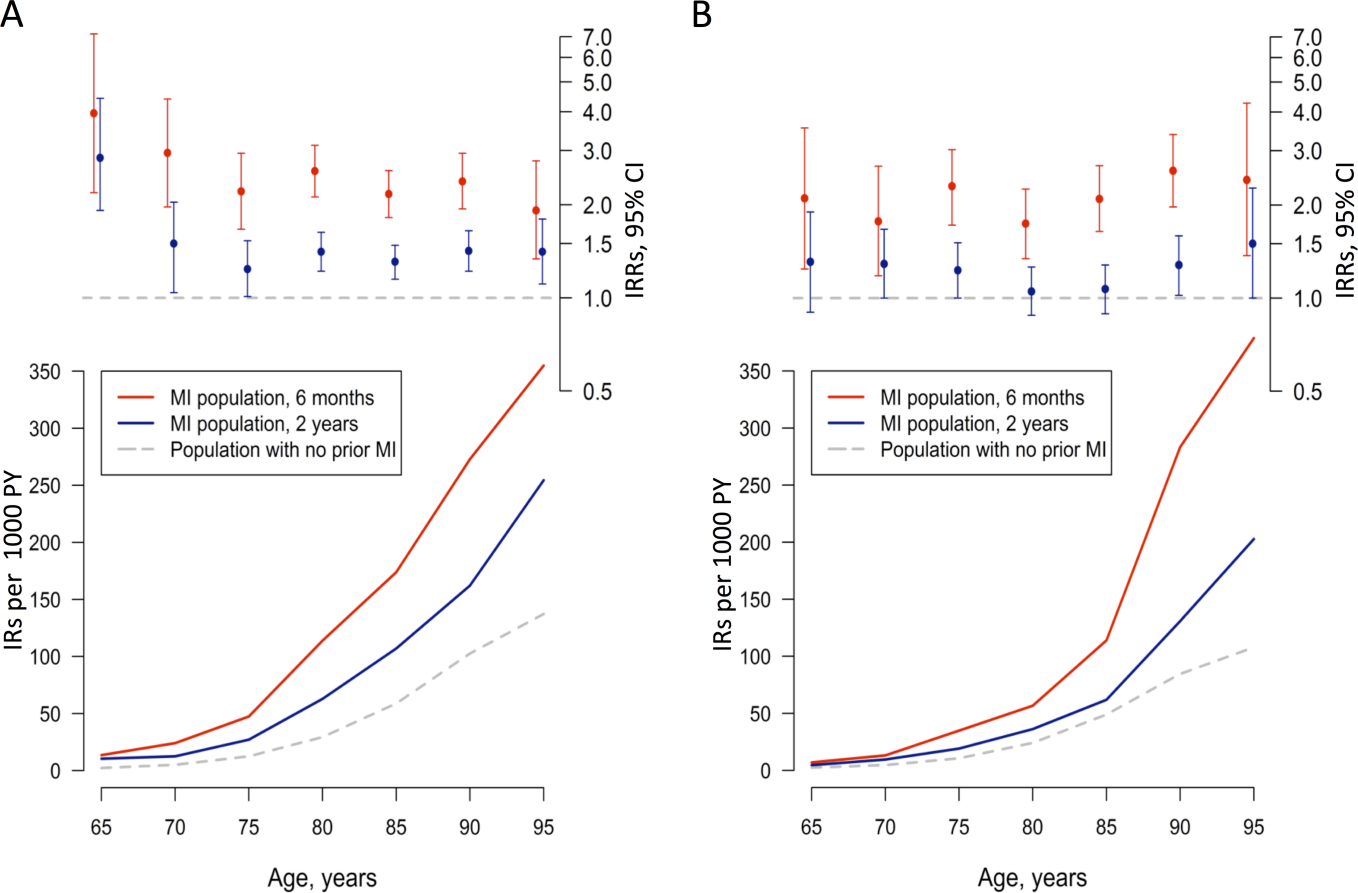
Age and sex-stratified incidence rates (IRs) and incidence rate ratios (IRRs) for nursing home admission. A) Women and B) Men. Red illustrates IRs and IRRs for nursing home admission within 6 months after discharge following myocardial infarction (MI), and blue illustrates IRs and IRRs within 2 years after discharge compared to the population with no prior MI (grey) (reference). IRs were calculated per 1000 person-year (PY). IRRs for nursing home admission were adjusted for calendar year, home care, living alone, baseline income, heart failure, stroke, arrhythmia, chronic kidney disease, diabetes, cancer, dementia, depression and Parkinson’s disease.

Within 6 months and 2 years after discharge following MI, 704 and 1,351 patients were admitted to nursing home, respectively. Crude IRs of nursing home admission increased with increasing age, both in patients with MI and in the population with no prior MI (Fig 1 and S3 Table). The 6 months IRs were higher than the 2 year IRs, but the latter were also significantly increased compared to the population with no prior MI. For 80-84-year-old women after MI, the 6 months and 2 years IRs were 113.9 and 62.9 compared to women with no prior MI of the same age 29.4. For 80-84-years-old men, these IRs were 56.0, 36.2, respectively, and for the male population with no prior MI of the same age the IR was 24.2. In adjusted analyses, which were similarly stratified on age and sex, the IRRs of nursing home admission were higher 6 months after discharge following MI compared to the population with no prior MI. While IRRs 2 years after discharge were increased for women the IRRs for men after 2 years showed a more varied pattern (Fig 1 and S4 Table). The 6 months and 2 years IRRs for 80-84-years-old subjects were 2.56 (95% CI 2.11–3.10) and 1.41 (95% CI 1.22–1.65) for women and 1.74 (95% CI 1.34–2.25) and 1.05 (95% CI 0.88–1.26) for men, respectively.

**Fig 2:**
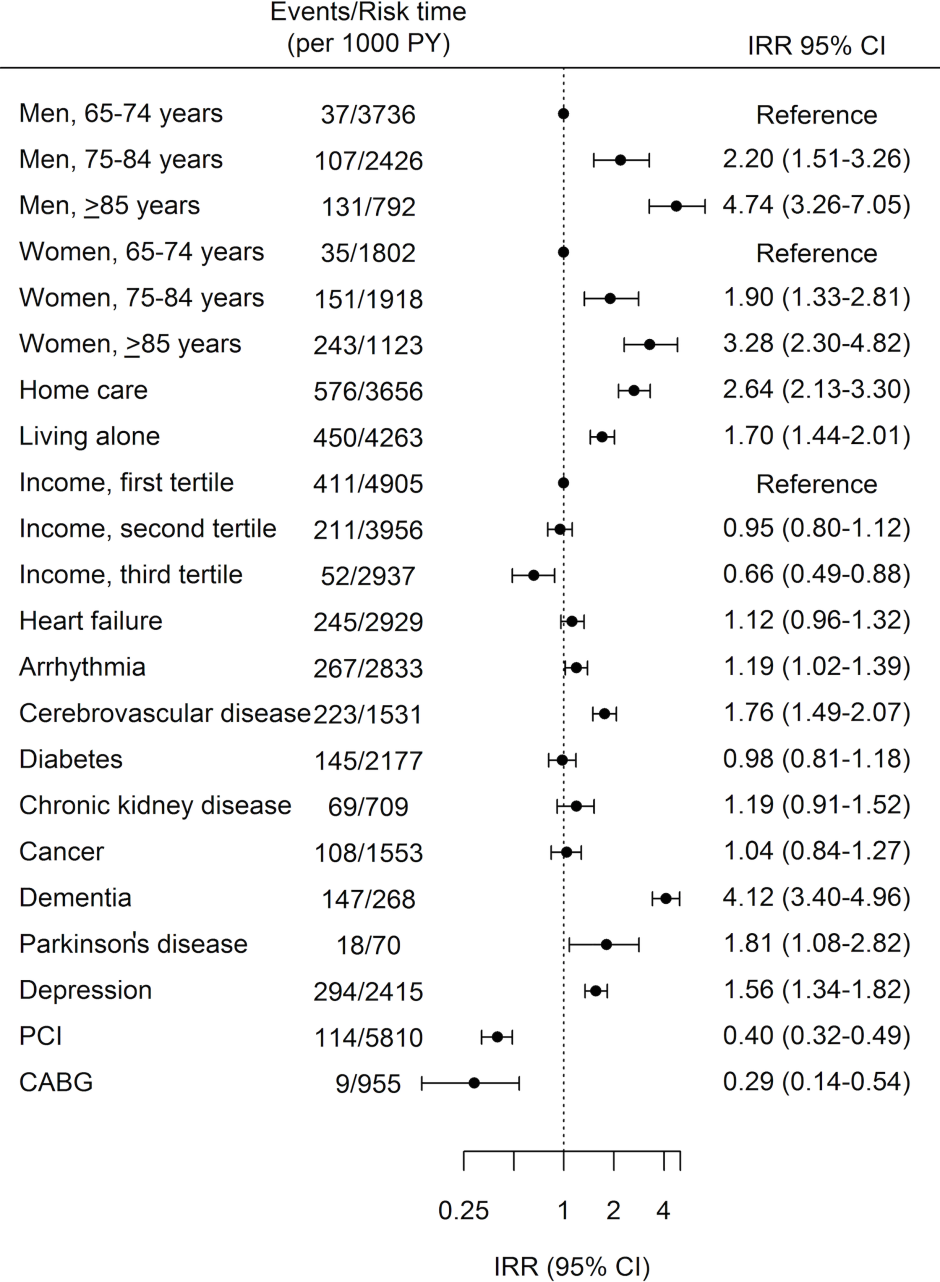
Risk factors for nursing home admission within 6 months after discharge following myocardial infarction. Incidence rate ratios (IRRs) with 95% confidence intervals (CIs) are displayed on a logarithmic scale. Sex-stratified estimates for age groups were adjusted with use of the variables displayed. Other estimates in the figure were adjusted for age modelled as a restricted cubic spline, sex and the other variables displayed. PCI; Percutaneous coronary intervention, CABG; Coronary artery by-pass grafting.

In the MI population, other predictors of nursing home admission within 6 months were dementia (IRR 4.12 [95% CI 3.40–4.96]), home care (IRR 2.64 [95% CI 2.13–3.30]), Parkinson’s disease (IRR 1.81 [95% CI 1.08–2.82]), cerebrovascular disease (IRR 1.76 [95% CI 1.49–2.07]), living alone (IRR 1.70 [95% CI 1.44–2.01]), depression (IRR 1.56 [95% CI 1.34–1.82]), and arrhythmia (IRR 1.19 [95% CI 1.02–1.39]) (Fig 2). Patients with high (third tertile) income (IRR 0.66 [95% CI 0.49–0.88]) compared to those with lowest (first tertile) income, or those who had undergone PCI (IRR 0.40 [95% CI 0.32–0.49]) or CABG (IRR 0.29 [95% CI 0.14–0.54]), respectively, were less likely to be resided in a nursing home. These overall results were similar in the sex- and age-stratified analyses (S5 Table). There were only minor differences between predictors for nursing home admission after 6 months and 2 years. However, after 2 years heart failure became a significant predictor (IRR 1.12 [1.00–1.26]); p = 0.04), while arrhythmia was no longer a predictor (IRRs 1.09 [0.97–1.22] (S1 Fig).

### Sensitivity analysis

**Fig 3:**
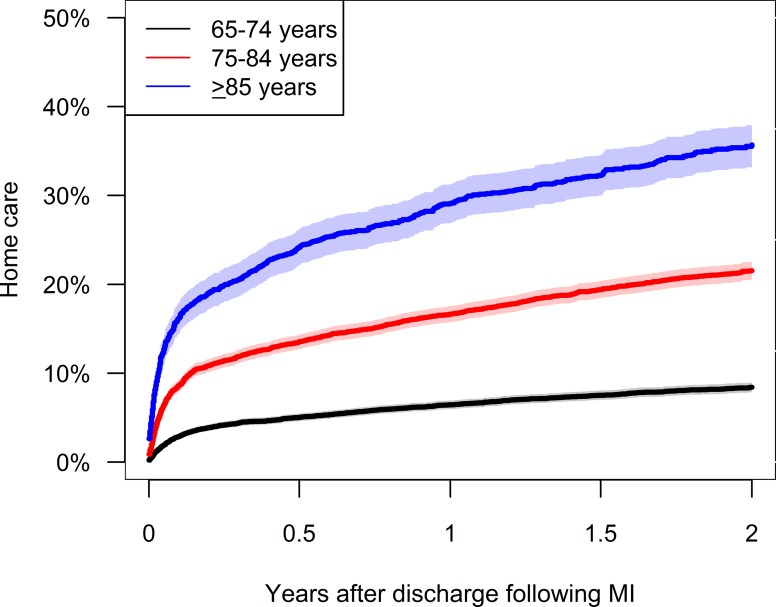
Cumulative incidence of home care provision after discharge following myocardial infarction (MI). Only patients who did not receive home care prior to hospitalisation were included.

In patients with MI who did not receive home care prior to MI (n = 19,114) the 6 months absolute risks of receiving home care after discharge were 5.0% (95% CI 4.6–5.5), 13.5% (95% CI 12.7–14.4), and 24.2% (95% CI 22.1–26.2) for the 65–74, 75–84, and ≥85-years-old, respectively (Fig 3). After 2 years these risks were 8.4% (95% CI 7.9–9.0), 21.5% (95% CI 20.5–22.5), and 35.7% (95% CI 33.3–38.1), respectively. Corresponding to Fig 1 the absolute risk of nursing home admission increased the most within 6 month after discharge and also increased with increasing age (S2 Fig).

## Discussion

In this nationwide registry-based study of more than 1.2 million individuals we found that the IRs of nursing home admission in elderly patients were more than 2-fold higher than that of the population with no prior MI 6 months after discharge from first-time MI for both sexes. This result was corroborated by adjusted analyses and 2 years after discharge the excess risk of nursing home admission was more prominent for women than men. Furthermore, important predictors of nursing home admission at six months were advanced age, dementia, home care, Parkinson’s disease, cerebrovascular disease, living alone, depression, and arrhythmia. These novel findings add importantly to the existing literature on circumstances of life and care dependency after MI.

In our study, the short-term (6 months) risk of nursing home admission after MI was higher than the risk after 2 years and short term risk may more directly reflect consequences of MI (hospitalisation) per se on functional status and care dependency better than the long-term risk. Additionally, current evidence shows that 3 out of 4 residents at nursing homes were hospitalised 6 months prior to nursing home admission [10]. Furthermore, our sensitivity analysis showed an increased need of home care in the period shortly after discharge in patients who lived at home after MI which is in agreement with studies that have demonstrated an increased need for home care, a decline in physical function, and loss of independence after discharge with MI [7,8,33]. Indeed, limited evidence has suggested that the functional capacity already declines prior to admission with MI potentially reflecting underlying coronary artery disease or other comorbidities [34,35].

The IRs for nursing home admission 6 months after discharge following MI in our study were significantly lower than findings in a German study (where IRs were higher after admissions with stroke, femoral fracture, and pneumonia, respectively, compared to MI) probably due to different study designs. These analyses were based on information from a routine data collection system from one health insurance company and included information on 4,266 community dwelling patients discharged after MI. Furthermore the analyses included only patients with a minimum hospital stay of 7 days thereby excluding less severe MI cases that probably also subsequently are less likely to be admitted to a nursing home [36].

A study by Luppa et al. found that prior MI was a predictor of nursing home admission, using direct interviews of 754 elderly without dementia from the population-based Leipzig Longitudinal Study of the Aged and in that study, the mean time to nursing home admission was 7.6 years after MI [15].

We found that dementia, Parkinson’s disease, cerebrovascular disease, depression, and arrhythmia were associated with nursing home admission. Indeed, dementia, Parkinson’s disease, and depression have previously been found to be predictors of nursing home admission in the general population and (for depression) in patients with coronary artery disease [11–13,15,16,37]. Living alone is also a well-described predictor of nursing home admission in the general population [11,13–15]. Patients with low income had a higher risk of nursing home admission than those with high income. Along this line, an American study found that uninsured patient had an increased risk of poor outcome after MI including independence loss and physical functional decline [8]. In our study, patients undergoing PCI or CABG were less likely to be admitted to nursing homes. In a previous study from Germany, CABG was associated with increased risk of disability at time of discharge after MI in likely agreement with the impact of major surgery and the opposite was the case for PCI [38]. However, although coronary artery revascularisation may contribute to increased functional capacity and self-dependency in the long term, patients selected for these procedures are generally less frail [39] indicating a substantial confounding by indication.

### Clinical implications

Population projections estimate a steep increase in the proportion of the elderly who have a high risk of MI and although these patients are getting older and more frail their mortality rates are decreasing [4,19]. In addition, ischemic heart disease is the leading contributor to disability-adjusted life years in individuals 60 years or older [20]. Admission to a nursing home is associated with great psychological concerns [9] and with an ageing population the societal expenditures are likely to increase. Therefore, the current novel findings are important by highlighting the increased risk of nursing home admission after MI in the elderly. More studies are needed to examine if increased awareness and treatment of functional decline after MI in the elderly can reduce the risk of nursing home admission in this vulnerable patient group.

### Strengths and limitations

The nationwide setting in a country where all citizens have equal access to healthcare, nursing home facilities, and home care provides optimal circumstances for obtaining real life results applicable to Denmark with minimal selection bias, but at the same time this setting limits the external validity of study results. The high validity of the MI diagnosis in the Danish National Patient Registry ensured a high accuracy of the inclusion criterion and the registry-based information on comorbidities and pharmaceutical drugs prevented recall bias. Our study was limited by the lack of clinical data that potentially contributed to residual confounding, e.g., measurements of activities of daily living, which is an important predictor for nursing home admission, as well as data on preclinical cognitive impairment [11,14,15]. Furthermore, although the study lacked data on functional decline, need of nursing home admission and provision of home care are likely to reflect impairment of overall health status.

Moreover, due to the registry-based design it is not possible to conclude that nursing home admission or need of home care were caused by MI, although the temporal pattern of higher IRs and IRRs in the early period after discharge from MI indicates a more definite causal relationship.

## Conclusion

In this nationwide study we found that the IRs of nursing home admission in elderly first-time MI patients were 2-fold higher than those of the population with no prior MI 6 months after discharge. In adjusted analyses, MI patients remained almost twice as likely to be admitted to a nursing home 6 months after discharge while after 2 years this excess risk was more prominent for women. Predictors of nursing home admissions were advanced age, dementia, home care, Parkinson’s disease, cerebrovascular disease, living alone, depression, and arrhythmia.

These novel findings inform on the vulnerability of elderly patients after MI and identify risk factors for nursing home admission that may be useful for identifying patients that need more attention during the post-MI rehabilitation process in order to be able to remain in their own home hereafter.

## Supporting information

S1 Table Classification codes used for definition of covariates. (DOCX)

S2 Table Sex- and age-stratified baseline characteristics of the myocardial infarction population. (DOCX)

S3 Table Age-stratified incidence rates corresponding to the estimates displayed in Fig 1. (DOCX)

S4 Table Age-stratified incidence rate ratio (IRR) as displayed in Fig 1. (DOCX)

S5 Table Sex and age-stratified estimates of nursing home admission within 6 months following myocardial infarction. The estimates are displayed as incidence rate ratio (IRR) and 95% confidence intervals (CI) and each estimate was adjusted for age and the other variables shown in the table.
(DOCX)

S1 Fig Risk factors for nursing home admission within 2 years after discharge following myocardial infarction. Incidence rate ratios (IRRs) with 95% confidence intervals (CIs) are displayed on a logarithmic scale. Sex-stratified estimates for age groups were adjusted with use of the variables displayed. Other estimates in the figure were adjusted for age modelled as a restricted cubic spline, sex and the other variables displayed.
(TIFF)

S2 Fig Cumulative Incidence of nursing home admission. (TIFF)
