# Supplementary material for: Nursing home admission after myocardial infarction in the elderly: A nationwide cohort study
Source: PLoS One. 2018 Aug 15;13(8):e0202177. doi: 10.1371/journal.pone.0202177 (PMC6093673; doi:10.1371/journal.pone.0202177)
Supplement: S1 Table — (DOCX) [file pone.0202177.s001.docx]

| **Comorbidities:** | **Specification:** |
| --- | --- |
| Myocardial infarction | ICD10: 121 |
| Heart failure | ICD10: I42, I50, I110, J81 |
| Arrhythmias | ICD10: Z950, I46-I49 |
| Hypertension | ICD10: I1 or combinations of two antihypertensive drugs reimbursed within 90 days. |
| Diabetes | ICD10: E10-E14  ATC: A10 |
| Chronic kidney disease | ICD10: 12-13, N03-N07, N08, N11, N14, N18, N25-N26, N28-N29, N158-N160, N162-164, N168, Z992, E102, E112, E132, E42 |
| Chronic obstructive pulmonary disease | ICD10: J42, J44 |
| Cerebrovascular disease | ICD10: G45-G46, H34, I60-I69 |
| Dementia | ICD10: G39,F00-F03  ATC: N06D |
| Depression | ICD10: F32-F33  ATC: N06A |
| Parkinson’s disease | ICD10: G20 |
| Cancer | ICD10: C00-C42, C44-C96 |
| **Medication:** | **ATC codes:** |
| Aspirin | B01AC06 |
| Statins | C10A |
| Beta-blockers | C07 |
| ACEIs/ARBs | C09 |
| Aspirin | B01AC06 |
| Statins | C10A |
| Beta-blockers | C07 |
| ACEIs/ARBs | C09 |
| Thienopyridines | B01AC04 and B01AC22 |
| Vitamin K antagonists | B01AA |
| Digoxin | C01AA05 |
| Thiazides | C03A |
| Calcium channel antagonists | C08 |
| Loop diuretics | C03C |
| Potassium-sparing diuretics | C03D |
| Ticagrelor | B01AC24 |
| **Invasive procedures:** |  |
| Coronary angiography | UXAC85 |
| Percutaneous coronary intervention | KFNG |
| Coronary artery bypass-grafting | KFNA, KFNC, KFND, KFNE |
| ICD; International classification of disease  ATC; Anatomical Therapeutic Chemical Classification | |

**S1 Table. Classification codes used for definition of covariates.**
