# Supplementary material for: Nursing home admission after myocardial infarction in the elderly: A nationwide cohort study
Source: PLoS One. 2018 Aug 15;13(8):e0202177. doi: 10.1371/journal.pone.0202177 (PMC6093673; doi:10.1371/journal.pone.0202177)
Supplement: S2 Table — (DOCX) [file pone.0202177.s002.docx]

**S2 Table. Sex- and age-stratified baseline characteristics of the myocardial infarction population.**

|  | Women | | | Men | | |
| --- | --- | --- | --- | --- | --- | --- |
| Age groups (years) | 65-74 | 75-84 | ≥85 | 65-74 | 75-84 | ≥85 |
| n | 3761 | 4333 | 2935 | 7833 | 5574 | 2103 |
| Age (median [IQR]) | 70 (68, 72) | 79 (77, 82) | 89 (86, 91) | 69 (67, 72) | 79 (77, 81) | 88 (86, 90) |
| Socioeconomic factors | | | | | | |
| Living alone (%) | 1800 (47.9) | 3100 (71.5) | 2705 (92.2) | 2187 (27.9) | 2078 (37.3) | 1123 (53.4) |
| Home care (%) | 598 (15.9) | 1800 (41.5) | 2231 (76.0) | 577 (7.4) | 1156 (20.7) | 1063 (50.5) |
| Income (%) |  |  |  |  |  |  |
| First tertile | 1782 (47.4) | 2834 (65.4) | 2067 (70.4) | 2363 (30.2) | 3273 (58.7) | 1380 (65.6) |
| Second tertile | 1268 (33.7) | 1184 (27.3) | 700 (23.9) | 2887 (36.9) | 1568 (28.1) | 531 (25.2) |
| Third tertile | 711 (18.9) | 315 (7.3) | 168 (5.7) | 2583 (33.0) | 733 (13.2) | 192 (9.1) |
| Comorbidities | | | | | | |
| Heart failure (%) | 514 (13.7) | 873 (20.1) | 678 (23.1) | 1050 (13.4) | 1056 (18.9) | 504 (24.0) |
| Arrhythmia (%) | 510 (13.6) | 944 (21.8) | 791 (27.0) | 1104 (14.1) | 1167 (20.9) | 536 (25.5) |
| Hypertension (%) | 1980 (52.6) | 2728 (63.0) | 1846 (62.9) | 3687 (47.1) | 3055 (54.8) | 1133 (53.9) |
| Diabetes (%) | 627 (16.7) | 748 (17.3) | 434 (14.8) | 1412 (18.0) | 1046 (18.8) | 307 (14.6) |
| Chronic kidney disease (%) | 133 (3.5) | 213 (4.9) | 153 (5.2) | 303 (3.9) | 390 (7.0) | 185 (8.8) |
| Cerebrovascular disease (%) | 319 (8.5) | 473 (10.9) | 380 (12.9) | 617 (7.9) | 657 (11.8) | 272 (12.9) |
| Peripheral artery disease (%) | 153 (4.1) | 254 (5.9) | 152 (5.2) | 313 (4.0) | 280 (5.0) | 105 (5.0) |
| COPD^a^ (%) | 498 (13.2) | 656 (15.1) | 273 (9.3) | 549 (7.0) | 705 (12.6) | 232 (11.0) |
| Dementia (%) | 25 (0.7) | 142 (3.3) | 165 (5.6) | 52 (0.7) | 126 (2.3) | 112 (5.3) |
| Depression (%) | 592 (15.7) | 757 (17.5) | 553 (18.8) | 595 (7.6) | 615 (11.0) | 255 (12.1) |
| Parkinson’s disease (%) | 14 (0.4) | 25 (0.6) | 15 (0.5) | 34 (0.4) | 49 (0.9) | 19 (0.9) |
| Cancer (%) | 329 (8.7) | 421 (9.7) | 241 (8.2) | 718 (9.2) | 847 (15.2) | 333 (15.8) |
| Medication | | | | | | |
| Aspirin (%) | 1007 (26.8) | 1528 (35.3) | 1302 (44.4) | 2174 (27.8) | 2058 (36.9) | 923 (43.9) |
| Statins (%) | 1340 (35.6) | 1512 (34.9) | 676 (23.0) | 2540 (32.4) | 1946 (34.9) | 528 (25.1) |
| Beta-blockers (%) | 946 (25.2) | 1341 (30.9) | 985 (33.6) | 1670 (21.3) | 1521 (27.3) | 596 (28.3) |
| ACEIs and ARBs (%) | 1473 (39.2) | 2009 (46.4) | 1283 (43.7) | 2886 (36.8) | 2323 (41.7) | 817 (38.8) |
| Calcium channel antagonists (%) | 818 (21.7) | 1230 (28.4) | 910 (31.0) | 1799 (23.0) | 1609 (28.9) | 580 (27.6) |
| Loop diuretics (%) | 480 (12.8) | 1008 (23.3) | 927 (31.6) | 698 (8.9) | 959 (17.2) | 588 (28.0) |
| Spironolactone (%) | 136 (3.6) | 215 (5.0) | 145 (4.9) | 216 (2.8) | 190 (3.4) | 95 (4.5) |
| Digoxin (%) | 100 (2.7) | 278 (6.4) | 317 (10.8) | 179 (2.3) | 287 (5.1) | 175 (8.3) |
| Vitamin K antagonists (%) | 160 (4.3) | 320 (7.4) | 208 (7.1) | 433 (5.5) | 518 (9.3) | 185 (8.8) |
| Invasive coronary procedures during index hospitalization | | | | | | |
| CAG | 2675 (71.1) | 2662 (61.4) | 894 (30.5) | 5799 (74.0) | 3648 (65.6) | 788 (37.5) |
| PCI | 1684 (44.8) | 1630 (37.6) | 631 (21.5) | 4530 (57.8) | 2566 (46.0) | 572 (27.2) |
| CABG | 70 (1.9) | 70 (1.6) | 4 (0.1) | 365 (4.7) | 189 (3.4) | 11 (0.5) |
| IQR:Interquartile range, COPD: Chronic obstructive pulmonary disease, ACEIs: Angiotensin-converting enzyme inhibitors, ARBs angiotensin receptor blockers, CAG: Coronary angiography, PCI: Percutaneous coronary intervention, CABG: Coronary by-pass surgery. | | | | | | |
