# Supplementary material for: Nursing home admission after myocardial infarction in the elderly: A nationwide cohort study
Source: PLoS One. 2018 Aug 15;13(8):e0202177. doi: 10.1371/journal.pone.0202177 (PMC6093673; doi:10.1371/journal.pone.0202177)
Supplement: S3 Table — (DOCX) [file pone.0202177.s003.docx]

**S3 Table. Age-stratified incidence rates corresponding to the estimates displayed in Figure 1.**

| **Age stratas** | **Events** | **Risk time per 1000 PY** | **Incidence rate^a^** | | **95% CI** |
| --- | --- | --- | --- | --- | --- |
| **Women, 6 months** | | | | | |
| 65-69 | 11 | 806 | 13.65 | | 7.56-24.64 |
| 70-74 | 24 | 996 | 24.09 | | 16.15-35.95 |
| 75-79 | 48 | 1014 | 47.31 | | 35.65-62.77 |
| 80-84 | 103 | 904 | 113.92 | | 93.91-138.19 |
| 85-89 | 124 | 712 | 174.03 | | 145.94-207.52 |
| 90-94 | 90 | 329 | 272.87 | | 221.94-335.49 |
| ≥95 | 29 | 81 | 355.98 | | 247.38-512.26 |
| **Women, 2 years** | | | | | |
| 65-69 | 25 | 2380 | | 10.50 | 7.10-15.54 |
| 70-74 | 41 | 3286 | | 12.47 | 9.19-16.94 |
| 75-79 | 92 | 3402 | | 27.04 | 22.04-33.17 |
| 80-84 | 188 | 2986 | | 62.96 | 54.57-72.63 |
| 85-89 | 243 | 2269 | | 107.08 | 94.43-121.43 |
| 90-94 | 173 | 1067 | | 162.05 | 139.62-188.09 |
| ≥95 | 66 | 259 | | 254.53 | 199.97-323.97 |
| **Women, general population** | | | | | |
| 65-69 | 2840 | 1,229,234 | | 2.31 | 2.23-2.40 |
| 70-74 | 4704 | 920428 | | 5.11 | 4.97-5.26 |
| 75-79 | 8410 | 672796 | | 12.50 | 12.24-12.77 |
| 80-84 | 13982 | 475419 | | 29.41 | 28.93-29.90 |
| 85-89 | 17437 | 296044 | | 58.90 | 58.03-59.78 |
| 90-94 | 12452 | 121446 | | 102.53 | 100.75-104.35 |
| ≥95 | 4281 | 31180 | | 137.30 | 133.24-141.47 |
| **Men, 6 months** | | | | | |
| 65-69 | 14 | 1996 | | 7.01 | 4.15-11.84 |
| 70-74 | 23 | 1739 | | 13.22 | 8.79-19.89 |
| 75-79 | 49 | 1403 | | 34.09 | 26.38-46.18 |
| 80-84 | 58 | 1022 | | 56.73 | 43.85-73.37 |
| 85-89 | 65 | 570 | | 114.02 | 89.41-145 |
| 90-94 | 54 | 190 | | 283.21 | 216.91-369.88 |
| ≥95 | 12 | 31.57 | | 380.07 | 215.85-669.25 |
| **Men, 2 years** | | | | | |
| 65-69 | 28 | 5954 | | 4.70 | 3.25-6.81 |
| 70-74 | 59 | 6119 | | 9.64 | 7.47-12.44 |
| 75-79 | 93 | 4850 | | 19.18 | 15.65-23.50 |
| 80-84 | 125 | 3450 | | 36.19 | 30.37-43.13 |
| 85-89 | 116 | 1902 | | 60.96 | 50.82-73.13 |
| 90-94 | 79 | 596 | | 132.54 | 106.31-165.24 |
| ≥95 | 23 | 117 | | 195.28 | 129.77-293.86 |
| **Men, general population** | | | | | |
| 65-69 | 2745 | 1,112,777 | | 2.47 | 2.38-2.56 |
| 70-74 | 3756 | 779101 | | 4.82 | 4.67-4.98 |
| 75-79 | 5465 | 516886 | | 10.57 | 10.30-10.86 |
| 80-84 | 7623 | 315555 | | 24.16 | 23.62-24-71 |
| 85-89 | 7588 | 154779 | | 49.03 | 47.93-50-14 |
| 90-94 | 4061 | 47991 | | 84.62 | 82.06-87.26 |
| ≥95 | 1069 | 9909 | | 107.87 | 101.60-114.54 |

PY:Person years; CI:Confidence intervals.

^a^ IRRs for nursing home admission were adjusted for calendar year, home care, living alone, baseline income, heart failure, stroke, arrhythmia, chronic kidney disease, diabetes, cancer, dementia, depression and Parkinson’s disease.
