# Supplementary material for: Nursing home admission after myocardial infarction in the elderly: A nationwide cohort study
Source: PLoS One. 2018 Aug 15;13(8):e0202177. doi: 10.1371/journal.pone.0202177 (PMC6093673; doi:10.1371/journal.pone.0202177)
Supplement: S4 Table — (DOCX) [file pone.0202177.s004.docx]

**S4 Table. Age-stratified incidence rate ratio (IRR) as displayed in Figure 1.**

| **Age stratas** | **IRR^a^** | **95% CI** |
| --- | --- | --- |
| **Women, 6 months** | | |
| 65-69 | 3.94 | 2.18-7.12 |
| 70-74 | 2.93 | 1.96-4.38 |
| 75-79 | 2.20 | 1.66-2.92 |
| 80-84 | 2.56 | 2.11-3.10 |
| 85-89 | 2.16 | 1.81-2.57 |
| 90-94 | 2.37 | 1.93-2.92 |
| ≥95 | 1.91 | 1.33-2.76 |
| **Women, 2 years** | | |
| 65-69 | 2.84 | 1.92-4.21 |
| 70-74 | 1.50 | 1.10-2.04 |
| 75-79 | 1.24 | 1.01-1.53 |
| 80-84 | 1.41 | 1.22-1.65 |
| 85-89 | 1.31 | 1.15-1.48 |
| 90-94 | 1.42 | 1.22-1.65 |
| ≥95 | 1.41 | 1.11-1.80 |
| **Men, 6 months** | | |
| 65-69 | 2.10 | 1.24-3.55 |
| 70-74 | 1.77 | 1.17-2.67 |
| 75-79 | 2.28 | 1.72-3.02 |
| 80-84 | 1.74 | 1.34-2.25 |
| 85-89 | 2.09 | 1.64-2.67 |
| 90-94 | 2.58 | 1.97-3.37 |
| ≥95 | 2.41 | 1.37-4.26 |
| **Men, 2 years** | | |
| 65-69 | 1.31 | 0.90-1.90 |
| 70-74 | 1.29 | 1.00-1.67 |
| 75-79 | 1.23 | 1.00-1.51 |
| 80-84 | 1.05 | 0.88-1.26 |
| 85-89 | 1.06 | 0.89-1.28 |
| 90-94 | 1.28 | 1.02-1.60 |
| ≥95 | 1.50 | 1.00-2.27 |
| CI; Confidence intervals.  ^a^ IRRs for nursing home admission were adjusted for calendar year, home care, living alone, baseline income, heart failure, stroke, arrhythmia, chronic kidney disease, diabetes, cancer, dementia, depression and Parkinson’s disease. | | |
