# Supplementary material for: Nursing home admission after myocardial infarction in the elderly: A nationwide cohort study
Source: PLoS One. 2018 Aug 15;13(8):e0202177. doi: 10.1371/journal.pone.0202177 (PMC6093673; doi:10.1371/journal.pone.0202177)
Supplement: S5 Table — The estimates are displayed as incidence rate ratio (IRR) and 95% confidence intervals (CI) and each estimate was adjusted for age and the other variables shown in the table. (DOCX) [file pone.0202177.s005.docx]

**S5 Table. Sex and age-stratified estimates of nursing home admission within 6 months following myocardial infarction.** The estimates are displayed as incidence rate ratio (IRR) and 95% confidence intervals (CI) and each estimate was adjusted for age and the other variables shown in the table.

|  | **IRR (95% CI)** | **IRR (95% CI)** |
| --- | --- | --- |
|  | **Women 65-74** | **Men 65-74** |
| Home care | 2.59 (1.21-5.54) | 4.39 (1.95-9.72) |
| Living alone | 1.57 (0.78-3.18) | 2.72 (1.31-5.63) |
| Income, second tertile | 0.96 (0.46-2.03) | 1.31 (0.62-2.81) |
| Income, third tertile | 0.50 (0.14-1.83) | 0.60 (0.20-1.83) |
| Heart failure | 1.19 (0.55-2.57) | 1.75 (0.89-3.44) |
| Cardiovascular disease | 1.15 (0.50-2.64) | 1.67 (0.80-3.45) |
| Arrhythmia | 0.56 (0.23-1.38) | 1.92 (0.94-3.92) |
| Chronic kidney disease | 1.86 (0.60-5.82) | 1.31 (0.48-3.58) |
| Diabetes | 2.38 (1.09-5.17) | 0.79 (0.37-1.68) |
| Cancer | 1.28 (0.48-3.38) | 0.90 (0.34-2.39) |
| Dementia | 31.15 (12.60-77.08) | 9.61 (3.91-23.60) |
| Depression | 1.66 (0.80-3.46) | 2.76(1.36-5.59) |
| Parkinson’s disease | 9.68 (2.19-42.83) | 1.24 (0.25-6.23) |
| PCI | 0.55 (0.25-1.22) | 0.66 (0.33-1.31) |

|  | **Women 75-84** | **Men 75-84** |
| --- | --- | --- |
| Home care | 3.32 (2.02-5.43) | 2.66 (1.65-4.30) |
| Living alone | 1.68 (1.18-2.41) | 1.97 (1.31-2.97) |
| Income, second tertile | 0.89 (0.62-1.27) | 0.71 (0.44-1.12) |
| Income, third tertile | 0.50 (0.22-1.14) | 0.91 (0.51-1.62) |
| Heart failure | 0.89 (0.63-1.27) | 1.07 (0.71-1.61) |
| Cardiovascular disease | 1.72 (1.20-2.46) | 2.11 (1.40-3.17) |
| Arrhythmia | 1.18 (0.85-1.65) | 1.29 (0.87-1.92) |
| Chronic kidney disease | 1.19 (0.67-2.13) | 0.75 (0.38-1.48) |
| Diabetes | 1.17(0.81-1.70) | 0.93 (0.58-1.48) |
| Cancer | 1.57 (1.05-2.35) | 0.74 (0.43-1.27) |
| Dementia | 3.25 (2.14-4.94) | 5.50 (3.45-8.76) |
| Depression | 1.62 (1.16-2.25) | 1.56 (1.04-2.34) |
| Parkinson’s disease | 0.86 (0.21-3.55) | 2.64 (1.04-6.72) |
| PCI | 0.27 (0.17-0.45) | 0.30 (0.18-0.50) |
|  |  |  |
|  | **Women ≥85** | **Men ≥85** |
| Home care | 1.16 (0.78-1.73) | 2.33 (1.41-3.83) |
| Living alone | 1.39 (1.03-1.90) | 2.04 (1.42-2.93) |
| Income, second tertile | 0.92 (0.69-1.23) | 1.19 (0.81-1.74) |
| Income, third tertile | 0.83 (0.49-1.38) | 0.54 (0.25-1.18) |
| Heart failure | 1.08 (0.82-1.43) | 1.50 (1.05-2.15) |
| Cardiovascular disease | 1.57 (1.17-2.10) | 2.23 (1.53-3.25) |
| Arrhythmia | 1.10 (0.84-1.44) | 1.56 (1.10-2.22) |
| Chronic kidney disease | 1.45 (0.90-2.34) | 1.29 (0.77-2.16) |
| Diabetes | 0.98 (0.69-1.40) | 0.62 (0.37-1.05) |
| Cancer | 0.71 (0.45-1.11) | 1.37 (0.91-2.08) |
| Dementia | 3.56 (2.56-4.96) | 5.13 (3.24-8.12) |
| Depression | 1.30 (0.99-1.70) | 1.64 (1.13-2.38) |
| Parkinson’s disease | 1.63 (0.58-4.57) | 1.30 (0.40-4.17) |
