# Supplementary figures and images for: Nursing home admission after myocardial infarction in the elderly: A nationwide cohort study
Source: PLoS One. 2018 Aug 15;13(8):e0202177. doi: 10.1371/journal.pone.0202177 (PMC6093673; doi:10.1371/journal.pone.0202177)

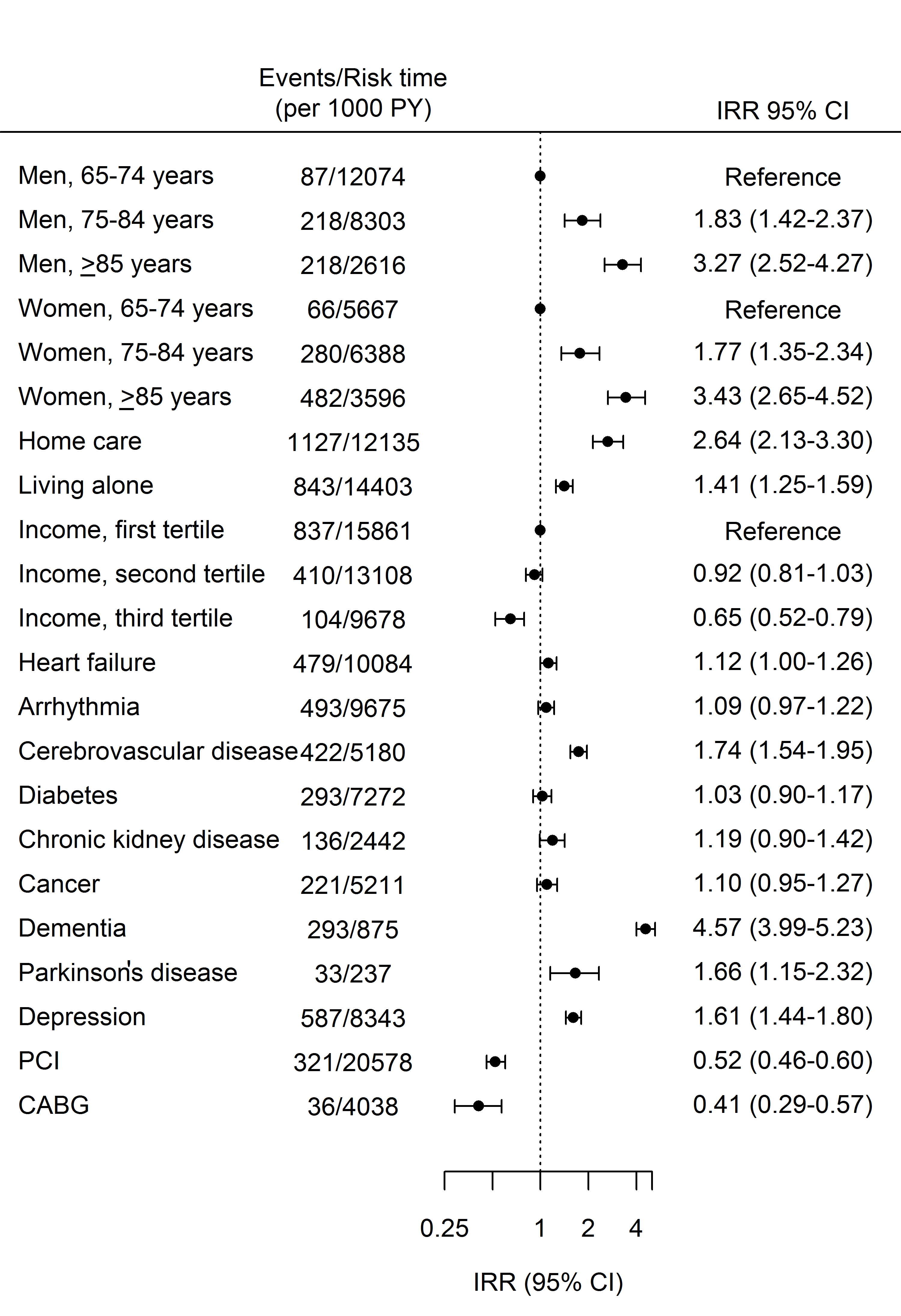

Supplement: S1 Fig — Incidence rate ratios (IRRs) with 95% confidence intervals (CIs) are displayed on a logarithmic scale. Sex-stratified estimates for age groups were adjusted with use of the variables displayed. Other estimates in the figure were adjusted for age modelled as a restricted cubic spline, sex and the other variables displayed. (TIFF) [file pone.0202177.s006.tiff]

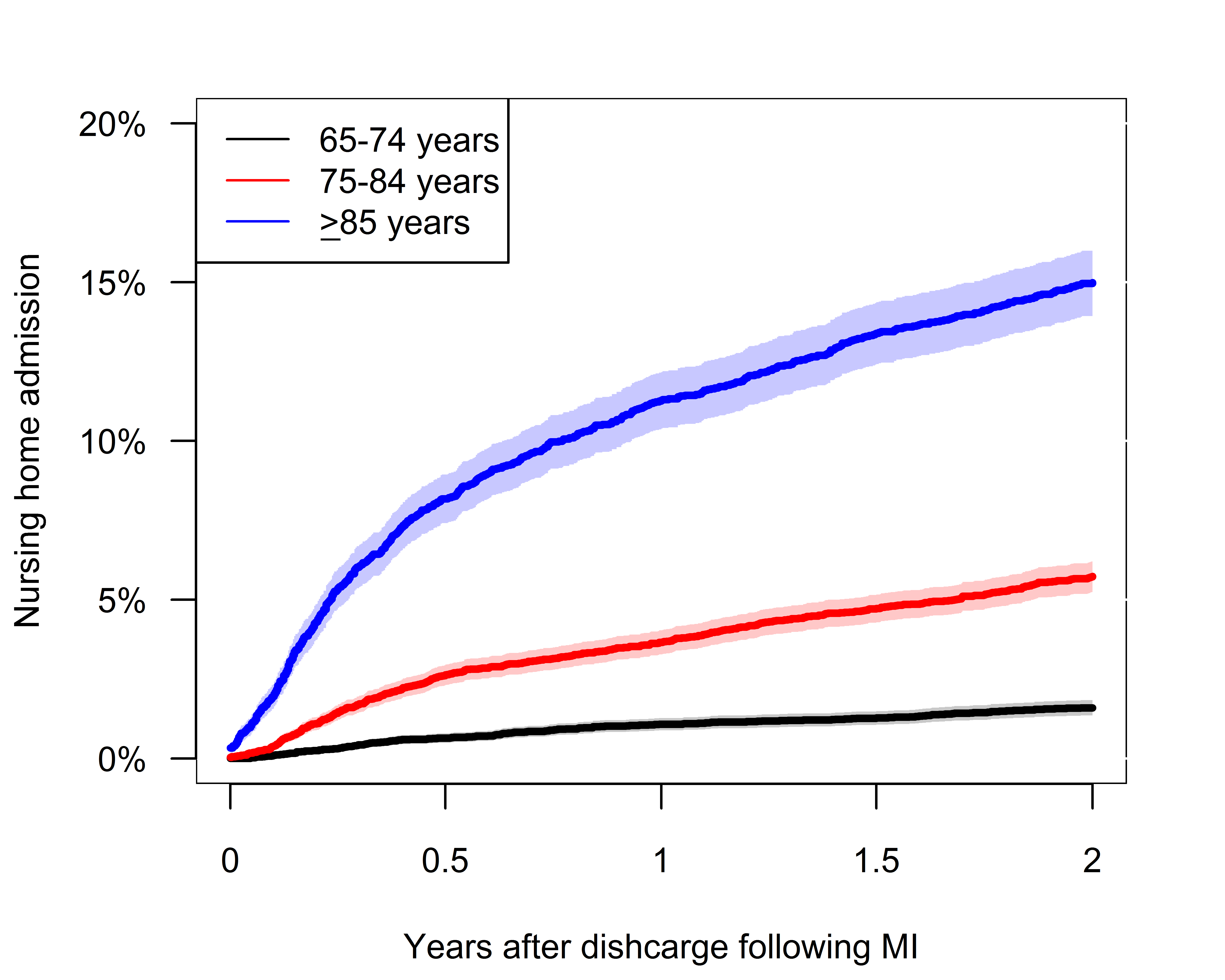

Supplement: S2 Fig — (TIFF) [file pone.0202177.s007.tiff]
